# Favourable outcomes for high‐risk Burkitt lymphoma patients (IPI 3‐5) treated with rituximab plus CODOX‐M/IVAC: Results of a phase 2 UK NCRI trial

**DOI:** 10.1002/jha2.3

**Published:** 2020-04-29

**Authors:** Elizabeth H. Phillips, Catherine Burton, Amy A. Kirkwood, Sharon Barrans, Anthony Lawrie, Simon Rule, Russell Patmore, Ruth Pettengell, Kirit M. Ardeshna, Silvia Montoto, Shankara Paneesha, Laura Clifton‐Hadley, David C. Linch, Andrew K. McMillan

**Affiliations:** ^1^ Division of Cancer Sciences the University of Manchester Manchester UK; ^2^ Cancer Research UK and UCL Cancer Trials Centre, UCL Cancer Institute University College London London UK; ^3^ HMDS, St James's University Hospital Leeds UK; ^4^ Plymouth University Medical School Plymouth UK; ^5^ Haematology Department, Castle Hill Hospital Hull UK; ^6^ Clinical Sciences Department, St George's University of London London UK; ^7^ Haematology Department University College Hospital London London UK; ^8^ Haemato‐oncology Department Barts Health NHS Trust London UK; ^9^ Haematology Department Heart of England NHS Trust Birmingham UK; ^10^ UCL Cancer Institute University College London London UK; ^11^ Haematology Department Nottingham University Hospitals NHS Trust Nottingham UK

**Keywords:** chemotherapy, CNS, HIV, immunotherapy, lymphomas, monoclonal antibodies

## Abstract

**Introduction:**

Outcomes after frontline treatment of Burkitt lymphoma (BL) have improved with the introduction of dose‐intense chemotherapy regimens, such as CODOX‐M/IVAC. While rituximab has increased survival rates for most forms of high‐grade B‐cell lymphoma, there has previously been hesitancy about incorporating it into BL treatment, partly due to concerns about increased toxicity. Prospective data using the standard dose CODOX‐M/IVAC regimen in combination with rituximab are lacking. We conducted a single‐arm phase 2 trial to assess the efficacy and toxicity of R‐CODOX‐M/R‐IVAC.

**Methods:**

Eligible patients were aged 18–65 years, with newly diagnosed BL with *MYC* rearrangement as the sole cytogenetic abnormality, and high‐risk disease, defined by an International Prognostic Index (IPI) score of 3‐5. Patients received two cycles of R‐CODOX‐M chemotherapy alternating with two cycles of R‐IVAC, followed by two further cycles of rituximab alone. The primary endpoint was 2‐year progression‐free survival.

**Results:**

Thirty‐eight patients were registered but after central pathology review, 27 patients had confirmed BL and commenced study treatment. Median age was 35 years, 14.8% patients had central nervous system involvement and 18.5% were HIV positive. Twenty‐two (81.4%) patients completed four cycles of chemotherapy. There were two treatment‐related deaths (7.4%). Two‐year progression‐free and overall survival rates were 77.2% (90% confidence interval [CI]: 56.0‐89.0) and 80.7% (90% CI: 59.6‐91.5), respectively.

**Conclusions:**

This prospective trial demonstrates excellent survival rates with R‐CODOX‐M/R‐IVAC in a high‐risk BL cohort. It provides reassuring evidence regarding the feasibility of this regimen and also provides a benchmark for future studies.

## 1 INTRODUCTION

Burkitt lymphoma (BL) is a rare and very aggressive form of B‐cell lymphoma, accounting for around 2% of all non‐Hodgkin lymphomas (NHL) in adults. BL is characterised by the presence of a germinal centre B‐cell phenotype, proliferation fraction approaching 100%, and t(8;14) or variant *MYC* rearrangement (*MYC‐*R) as the sole cytogenetic abnormality [1, 2, 3]. Atypical features are often present, making the differential diagnosis between BL and other high‐grade B‐cell lymphomas (HGBL) challenging. Clinically, BL is a rapidly progressive tumour with high rates of extranodal involvement and a propensity to spread to the central nervous system (CNS) [4, 5]. Although high cure rates can be achieved, frontline treatment usually offers the only opportunity for disease cure, with very poor outcomes for patients with relapsed or refractory disease [6, 7].

Survival rates have improved markedly over recent decades [8, 9], largely through the introduction of dose‐intense chemotherapy regimens incorporating both CNS penetrating drugs and hyperfractionated alkylating agents [1, 10–12]. Most protocols include rapid cycling of multiple non‐cross resistant and cell cycle–specific agents, which are administered as soon as haematological recovery occurs to prevent re‐emergence of resistant disease clones. The Magrath regimen, consisting of alternating courses of CODOX‐M and IVAC chemotherapy, encompasses these principles and has resulted in encouraging survival rates in a number of BL studies [1, 13, 14, 15]. Several modifications to the original regimen, including a reduction in methotrexate and vincristine doses, have improved tolerability without compromising efficacy [1]. Despite these advances, around one‐third of adult patients will progress after frontline CODOX‐M/IVAC or similar dose‐intense chemotherapy regimens [1, 9] and improvements are still needed to reduce failure rates further.

The addition of the anti‐CD20 antibody rituximab to standard chemotherapy regimens has considerably improved outcomes and is considered standard of care for most forms of B‐cell NHL [16, 17]. BL has high levels of CD20 expression and rituximab can induce direct cell death in BL cells in vitro [18]. However, concerns about potential myelotoxic and immunosuppressive effects of rituximab have delayed the adoption of immunochemotherapy into clinical practice for BL. Indeed, it is only recently that randomised trials have demonstrated that rituximab improves outcomes in BL, when combined with the intensive Lymphome Malin B (LMB) regimen [19]. A preliminary study of rituximab in combination with the intensive hyper‐CVAD regimen in BL suggested that the addition of rituximab can improve event‐free survival by as much as 28%, when compared with historical controls [20]. In light of these early encouraging results, we conducted a prospective, multi‐centre trial to assess the efficacy and toxicity of rituximab in combination with CODOX‐M/IVAC in a high‐risk cohort of BL patients.

## 2 METHODS

**FIGURE 1:**
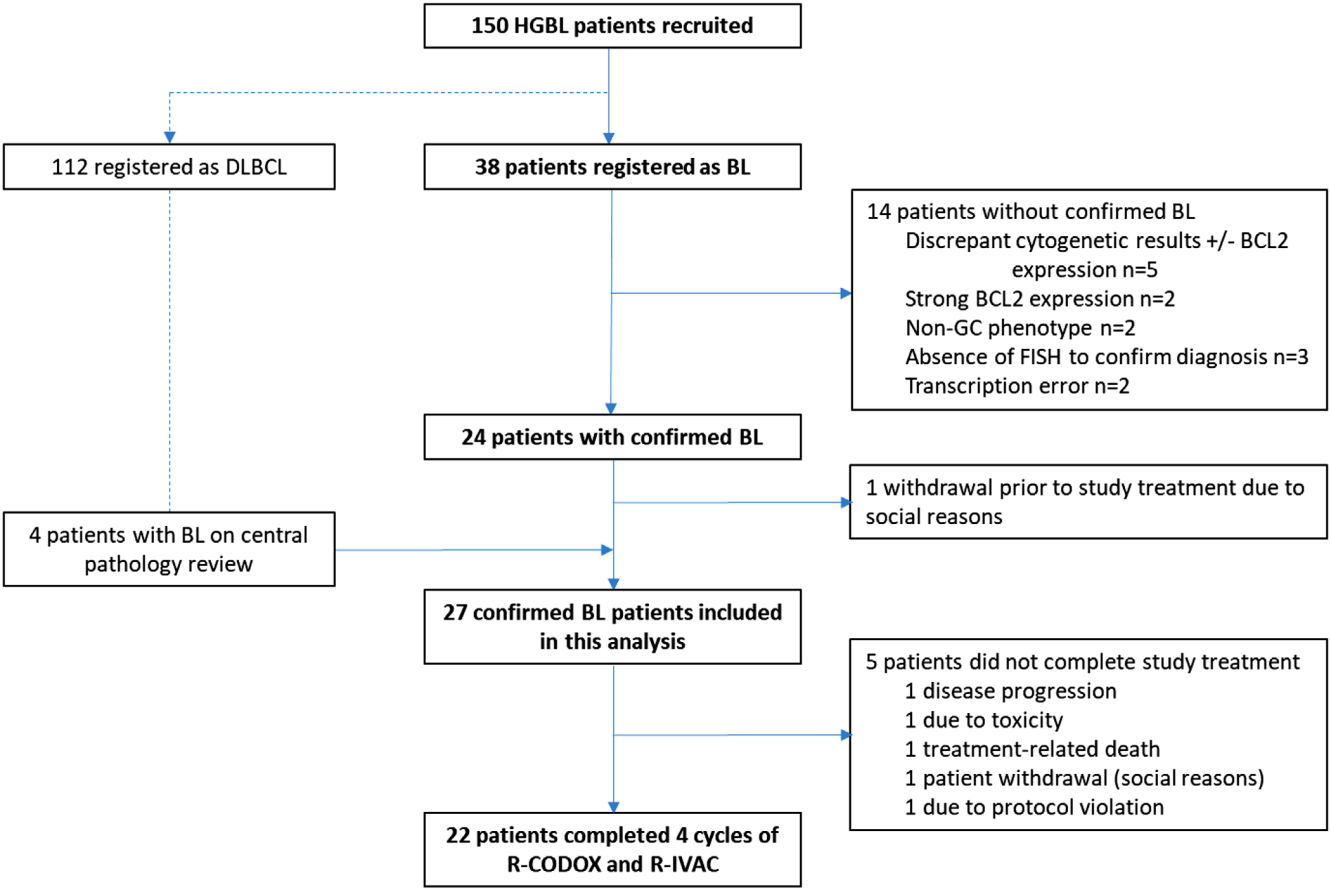
Consort diagram

The phase 2 R‐CODOX‐M trial included two parallel treatment cohorts assessing the same treatment regimen in different types of HGBL. Outcomes for patients in the BL cohort are reported here; the diffuse large B‐cell lymphoma (DLBCL) cohort will be reported separately. This design permitted crossover of patients between the two trial arms following central pathology review, which facilitated the inclusion of a uniform diagnostic cohort of confirmed BL patients (see Figure 1).

### 2.1 Eligibility

Eligible patients were aged 18‐65 years with stage II‐IV, previously untreated CD20^+^ HGBL and an International Prognostic Index (IPI) score of 3‐5 [21]. BL was defined as HGBL with a germinal centre phenotype, absent BCL‐2 expression, high proliferation rate (>90%) and the presence of a *MYC*‐R, without *BCL2* or *BCL6* rearrangement, consistent with current WHO guidelines [3]. Original diagnostic material was centrally reviewed by the Leeds Haematological Malignancy Diagnostic Service.

Inclusion criteria included adequate liver, renal, cardiac and bone marrow function, unless directly attributable to disease infiltration. Performance status (PS) was permissive. In light of emergent data demonstrating feasibility of dose‐intense immunochemotherapy in HIV‐positive patients with NHL, a protocol amendment allowed inclusion of HIV‐positive patients provided that there was no prior history of opportunistic infection, PS was ≤2 and baseline CD4 count was ≥100 cells/mm^3^. Baseline investigations included bone marrow biopsy, contrast‐enhanced CT of the neck to pelvis and cerebrospinal fluid cytology ± MRI. Informed consent was obtained from all patients prior to trial entry according to the Declaration of Helsinki.

### 2.2 Study treatment

There was no pre‐phase chemotherapy, although prior administration of corticosteroids for up to 10 days was permitted. All patients received either allopurinol or rasburicase before treatment, according to local practice. Treatment consisted of two cycles of CODOX‐M (cyclophosphamide, vincristine, doxorubicin, methotrexate) alternating with two cycles of IVAC (ifosfamide, etoposide, cytarabine), as previously described [1]. Rituximab (375 mg/m^2^) was administered concurrently on day 1 of each cycle, with additional doses on day 11 of CODOX‐M and days 21 and 42 after the final IVAC cycle. Standard treatment included eight intrathecal chemotherapy injections (four methotrexate and four cytarabine); patients with CNS involvement received an additional four doses of intrathecal chemotherapy. The interval between consecutive cycles was determined by haematological recovery, commencing as soon as neutrophils were >1 × 10^9^/L and platelets >75 × 10^9^/L. Mandatory supportive care included pegylated granulocyte colony stimulating factor (G‐CSF), herpes simplex, and *Pneumocystis jirovecii* prophylaxis.

End‐of‐treatment response was assessed 4 weeks after completion of chemotherapy using contrast‐enhanced CT according to international guidelines [22]. The study design predated widespread availability of ^18^fluorodeoxyglucose positron emission tomography (PET) for response assessment but was encouraged to assess residual masses. Radiotherapy was not included in this trial but was permitted for those with initial disease bulk or CNS disease, and for residual PET‐positive disease.

### 2.3 Endpoints and statistical methods

The primary endpoint was the progression‐free survival (PFS) rate at 2 years. Secondary endpoints included complete response (CR) rate, overall survival (OS) and toxicity.

Using a Fleming design, recruiting at least 30 BL patients would give 80% power to show an improvement of 20% at 2 years (from 65% to 85%) with a 1‐sided 5% alpha. The trial was also open to high‐risk DLBCL patients (95 required) and, given that the LY10 trial had noted that as many as 50% of patients with highly proliferative HGBL were not true BL [1], we therefore assumed that a reasonable number may switch cohorts after central review, and a total target of 150 was fixed. Recruitment was to be stopped once this target was reached, irrespective of the number of BL patients treated.

PFS was calculated as the time from registration until either disease progression or death, on an intention‐to‐treat basis, however those found to be ineligible after registration or those who withdrew before starting treatment (for reasons unrelated to their disease) were excluded. Patients who were alive and progression‐free were censored at the date last seen. All analyses were performed using Stata v15.1 (Stata Corp, TX).

## 3 RESULTS

### 3.1 Patient registration and pathology results

A total of 150 patients with HGBL were recruited at 36 UK sites between September 2009 and March 2013, of which 38 were registered as BL (Figure 1). Diagnostic tissue for central pathology review was available in 30 patients (78.9%); pathology reports issued by specialist haematopathology centres were reviewed for all other patients. The diagnosis of BL could only be confirmed in 24 patients (63.2%), one of whom withdrew immediately after registration for social reasons and has been excluded from all analyses. Fourteen patients were revised to a diagnosis of DLBCL or HGBL, due to disagreement between central and local pathology review (*n* = 9; 23.7%), administrative errors (*n* = 2) or absence of fluorescence in situ hybridisation to confirm *MYC‐*R (*n* = 3). An additional four patients initially registered as DLBCL had a centrally confirmed diagnosis of BL and have been included in this analysis.

In total, 27 BL patients were included in this study. Baseline patient characteristics are shown in Table 1. CNS disease was present in four patients (14.8%), although only CSF cytology, but not immunophenotyping, was mandated, therefore low‐level subclinical leptomeningeal involvement could not be excluded.

**TABLE 1:** Baseline characteristics

Baseline characteristic	N = 27
Age (years), median (range)	35 (20‐64)
Age, N (%)	
Under 40	15 (55.6)
40‐60	10 (37.0)
60 and over	2 (7.4)
Sex, N (%)	
Female	3 (11.1)
Male	24 (88.9)
ECOG performance status, N (%)	
0	6 (22.2)
1	8 (29.6)
2	8 (29.6)
3	5 (18.5)
Stage, N (%)	
III	3 (11.1)
IV	24 (88.9)
IPI score, N (%)	
3	14 (51.9)
4	13 (48.1)
B symptoms, N (%)	
Absent	12 (44.4)
Present	15 (55.6)
CNS disease baseline, N (%)	
No	23 (85.2)
Yes	4 (14.8)
Bone marrow involvement, N (%)	
No	10 (37.0)
Yes	14 (51.9)
Unknown	3 (11.1)
HIV status, N (%)	
Negative	22 (81.5)
Positive	5 (18.5)
Elevated LDH, N (%)	
Yes	27 (100.0)
More than one extra nodal site, N (%)	
No	3 (11.1)
Yes	24 (88.9)
Central pathology review, N (%)	
Yes	23 (85.2)
Review of local pathology results	4 (14.8)
Confirmed *MYC* rearrangement, N (%)	
Yes	27 (100)

Abbreviations: CNS, central nervous system; ECOG, Eastern Co‐operative Oncology Group; HIV, human immunodeficiency virus; IPI, international prognostic index; LDH, serum lactate dehydrogenase.

### 3.2 Study treatment

Twenty‐two patients (81%) completed all four cycles of alternating R‐CODOX‐M and R‐IVAC. Reasons for early treatment discontinuation were toxicity (*n* = 2), disease progression (*n* = 1) and non‐clinical (*n* = 2; Figure 1). The first rituximab dose was given with day 1 of CODOX‐M chemotherapy in 16 (61.5%) patients, and within 48 hours of CODOX‐M administration in 20 (76.9%) patients. The median interval between chemotherapy cycles was 26.5 days for cycles 1‐2 (range 19‐41), 21.5 days for cycles 2‐3 (range 15‐38) and 28.5 days for cycles 3‐4 (range 19‐49). There was no evidence that the addition of rituximab delayed count recovery and increased treatment intervals. Equivalent median cycle lengths from the preceding LY10 study, which used the same chemotherapy backbone without rituximab, were as follows: 27 days for cycles 1‐2, 21 days for cycles 2‐3 and 29 days for cycles 3‐4 [1]. Only two patients (7.4%) received consolidation radiotherapy.

### 3.3 Toxicity

Details of all grade 3‐5 toxicities are presented in **Table** **2**. Significant haematological toxicity occurred, as expected with this dose intense regimen, with all patients experiencing grade 3‐4 haematological toxicity and 66.7% with a grade ≥3 infection. Only one patient, who did not receive rituximab with treatment initiation, developed grade 3 tumour lysis. There was one early treatment‐related death due to infection in a 51‐year old patient with a PS of 3. One other patient died due to secondary acute myeloid leukaemia. All five HIV‐positive patients completed protocol treatment without evidence to suggest excess toxicity; only two of these patients experienced grade ≥3 infection, noting that inclusion criteria were different for those with HIV (PS < 2).

**TABLE 2:** Grade 3‐5 adverse events according to the Common Toxicity Criteria for Adverse Events (CTCAE) v3

System organ class/adverse event	N (%)
Blood and bone marrow	27 (100.00)
Anaemia	6 (22.22)
Bone marrow suppression	1 (3.70)
Leukopaenia	1 (3.70)
Neutropaenia	27 (100.00)
Thrombocytopaenia	25 (92.59)
Cardiac	3 (11.11)
Cardiac (not otherwise specified)	1 (3.70)
Hypotension	2 (7.41)
Constitutional	10 (37.04)
Fatigue	2 (7.41)
Fever	8 (29.63)
Gastrointestinal	12 (44.44)
Anorexia	1 (3.70)
Diarrhoea	4 (14.81)
Mucositis	9 (33.33)
Nausea	2 (7.41)
Vomiting	2 (7.41)
Infection	19 (70.37)
Febrile neutropaenia	6 (22.22)
Infection	19 (70.37)^*^
Laboratory/metabolism	4 (14.81)
Hypokalaemia	1 (3.70)
LFTs	2 (7.41)
Lymphatic	1 (3.70)
Oedema	1 (3.70)
Neurology	1 (3.70)
Cognitive disturbance	1 (3.70)
Personality changes	1 (3.70)
Pain	3 (11.11)
GI pain	2 (7.41)
Musculoskeletal pain	1 (3.70)
Neurology headache	1 (3.70)
Pulmonary/upper respiratory	2 (7.41)
Pulmonary oedema	1 (3.70)
Pleural effusion	1 (3.70)
Secondary malignancy	1 (3.70)
Acute myeloid leukaemia	1 (3.70)^*^
Syndromes	1 (3.70)
Tumour lysis	1 (3.70)
Any non‐haematological	24 (88.89)

Abbreviation: LFTs, liver function tests.

* One grade 5 event.

### 3.4 Patient outcomes

Overall response rate was 85.2%, with 21 patients (77.8%) achieving CR or unconfirmed CR (CRu) and two partial responses (7.4%). One patient had progressive disease during treatment and three patients were not evaluable due to study withdrawal (*n* = 2) or death (*n* = 1).

**FIGURE 2:**
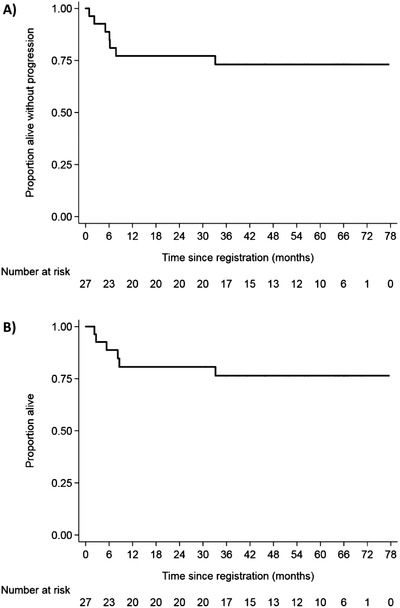
Outcomes for Burkitt lymphoma patients: (A) progression‐free survival and (B) overall survival

After a median follow‐up of 56.9 months (range 2.2‐77.5), 2‐year PFS was 77.2% (90% CI: 60.1‐87.6) and 2‐year overall survival was 80.7% (90% CI: 63.8‐90.3) (Figure 2). Five patients had disease progression, one of whom was lost to follow‐up after failing to respond to initial salvage therapy. No progressions have been reported beyond 2 years but there was one late death due to secondary malignancy at 32 months. Six deaths occurred in total, due to BL (*n* = 3), study treatment (*n* = 2) or salvage chemotherapy (*n* = 1).

**FIGURE 3:**
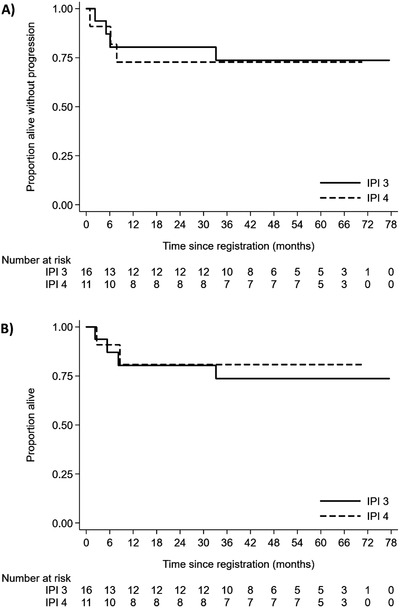
Progression‐free survival (A) and overall survival (B) according to IPI score

**FIGURE 4:**
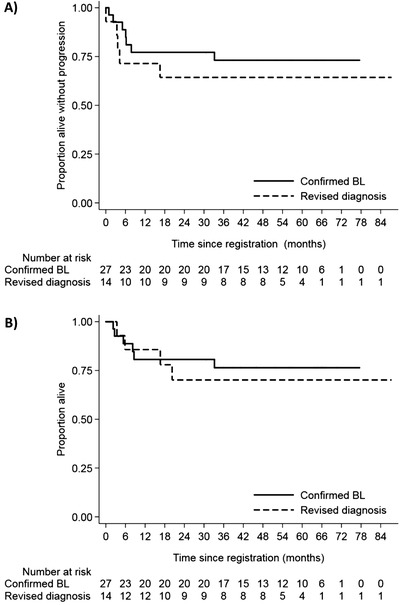
Outcomes for patients according to central pathology review: progression‐free (A) and overall survival (B) for patients with a revised diagnosis of DLBCL/HGBL and those with confirmed BL

Two‐year PFS rates for patients with an IPI score of 3 and 4 were 79.9% (90% CI: 54.2‐91.3) and 75.0% (90% CI: 47.4‐89.5), respectively (Figure 3). Of four patients with CNS disease at registration, one has relapsed with synchronous systemic and CNS disease. All five HIV‐positive patients are alive and progression free. Outcomes for 14 patients that had their initial diagnosis of BL revised to HGBL or DLBCL after central review were slightly lower than for the confirmed BL cohort, with 2‐year PFS and OS rates of 64.3% (90% CI: 39.6‐81.0) and 70.1% (90% CI: 44.3‐85.7), respectively (Figure 4).

## 4 DISCUSSION

Although the addition of rituximab to CODOX‐M/IVAC is considered standard of care for high‐risk BL in a number of countries worldwide [23, 24], it was adopted into routine care without any direct clinical trial evidence in this patient group. This is the first trial to prospectively assess the efficacy of standard CODOX‐M/IVAC with rituximab. Two‐year PFS with this regimen was 77.2% (90% CI: 60.1‐87.6), which compares favourably to a PFS of 60‐65% for high‐risk BL patients treated in the LY10 trial with CODOX‐M/IVAC alone, without rituximab [1]. Notwithstanding the inherent limitations in making comparisons between studies, an apparent improvement in PFS was observed in our trial despite recruitment of higher risk patients (IPI score 3‐5) than in LY10 (IPI score ≥ 2). Other studies have also shown improved outcomes with rituximab in BL [24–26]. A randomised phase 3 trial demonstrated a 13% improvement in PFS with rituximab for BL patients treated with the dose‐intense LMB regimen [19]. We considered using a randomised trial design, including an arm without rituximab, but felt this would lack equipoise in light of the clear survival benefit with rituximab in DLBCL [16, 27] and excellent preliminary results in BL [20]. Our results add to the growing body of evidence that immunochemotherapy should be considered standard of care in BL.

One limitation of this study is the sample size. We did not meet the 2‐year PFS rate of ≥85% we hoped for, with the 90% confidence interval (60.2‐87.6%) unable to exclude the lower limit of 65%. We had aimed for 30 patients, but recruited 27 with confirmed BL, due to high rates of crossover after central pathology review. However, the main reason that we did not meet our endpoint is that our initial prediction of a 20% improvement in PFS, based on a historical comparison, was over‐optimistic [20]. Although the trial was not powered to demonstrate smaller improvements, our observed PFS rate (77.2%) still represents a clinically meaningful improvement and is consistent with the magnitude of benefit seen with rituximab in other studies [19].

Evidence for use of R‐CODOX‐M/R‐IVAC has largely been based on retrospective studies, which report PFS rates of 74‐81% and OS rates of 72‐77% [24, 25, 28, 29]. These studies were heterogeneous in their inclusion criteria, lack central pathology review and cannot accurately gauge toxicity in retrospect. Two single‐arm prospective studies have assessed variations of the CODOX‐M/IVAC regimen with rituximab. Evens et al investigated liposomal doxorubicin and a non‐standard rituximab dose (500 mg/m^2^) with CODOX‐M/IVAC in 20 high‐risk patients. Two‐year PFS was similar to our trial (76%), but cardiac toxicity was significant [30]. The AMC048 study assessed R‐CODOX‐M/R‐IVAC in a high‐risk, HIV‐positive population (*n* = 34), incorporating four doses of rituximab (375 mg/m^2^) but with altered dose density and non‐hyperfractionated cyclophosphamide; 1‐year PFS was 69% (51‐82) [31]. Results of our trial prospectively confirm that the standard R‐CODOX‐M/R‐IVAC regimen is an effective option for frontline treatment of high‐risk BL.

The optimum frontline regimen for treatment of BL remains unclear. No trials comparing modern immunochemotherapy regimens have been published to date and comparison between trials is precluded by variation in clinical and pathology inclusion criteria. Definitions of ‘high‐risk’ BL vary, although trials most require only one of elevated LDH, PS ≥2, stage 3–4 disease and bulky disease, and fail to discriminate prognosis for the 80–90% of BL patients identified as ‘high risk’ [30, 32]. The IPI has consistently been identified as a prognostic measure in BL, along with age and PS [4, 26, 33, 34]. Indeed, a large, recent BL real‐world data study showed that other high‐risk features, such as CNS involvement, were associated with IPI and failed to retain independent prognostic significance after correction for IPI [35]. Our BL cohort, defined by an IPI score ≥3 and with 48% PS ≥2, is therefore higher risk than most ‘high‐risk’ BL studies, even though patients were relatively young (median age of 36 years).

Our outcomes are broadly in line with IPI 3‐5 patients receiving dose‐intense immunochemotherapy regimens in other trials, all of which include hyperfractionated cyclophosphamide and CNS‐penetrating agents. The GMALL B‐NHL2002 trial reported a 4‐year OS of 75% for patients with an IPI score of 3‐5 [4]. The CALGB10002 study reported 4‐year OS of 72% for IPI score 3 and 55% for IPI score 4‐5 [34]. Very good survival rates have been achieved in trials of lower intensity dose‐adjusted EPOCH‐R therapy, although these include patients with a higher median age but otherwise lower clinical risk profile than our trial, therefore comparison between regimens is not possible [32]. One advantage of R‐CODOX‐M/R‐IVAC is the shorter duration of chemotherapy: 14 weeks, compared with 18–26 weeks for other high‐risk BL regimens [4, 19, 34, 36]. Notably, a recent retrospective study that demonstrated a favourable profile with respect to cost and treatment duration for R‐CODOX‐M/R‐IVAC compared with other intensive BL regimens [37].

Toxicity in our study was largely manageable, demonstrating that CODOX‐M/IVAC with rituximab is safe and feasible in high‐risk BL. Even though most patients had widespread, advanced‐stage disease, full dose intensity and concurrent administration of rituximab with R‐CODOX‐M was deliverable from day 1 in most patients, with only one instance of tumour lysis. Only two patients (7.4%) discontinued treatment for toxicity‐related reasons, which support real‐world data showing high completion rates with R‐CODOX‐M/IVAC [37]. Many regimens include a short chemotherapy pre‐phase to reduce tumour burden, although this trial and other studies suggest that this may not be required in most cases [24]. We found no evidence to suggest that toxicity with rituximab in this trial was higher than with CODOX‐M/IVAC alone. In particular, haematological recovery, reflected in the length of each treatment cycle, was very similar in our trial to the previous LY10 trial [1]. G‐CSF was administered in both studies, although in pegylated form in this study. It is important to note, however, that both LY10 and the DLBCL arm of this trial identified higher toxicity rates in older patients, therefore this intensive regimen is only applicable to fit older patients with a relatively good PS [38].

A strength of this trial was the use of central pathology review to determine allocation to either the BL or DLBCL/HGBL trial arms, thus identifying a uniform cohort with confirmed BL. Diagnostic discrepancy rates for BL were high: 11 of 36 patients (30.6%) registered as BL switched to DLBCL/HGBL after central review. Larger BL trials have identified non‐BL diagnoses in 10‐21% of patients on central review [15, 19, 34]. Even specialist haematopathologists may be unable to reach diagnostic consensus in up to 35% of BL cases [39]. This highlights the challenge in differentiating BL from other forms of HGBL in clinical practice, and the importance of comprehensive central pathology review in BL studies. Nevertheless, R‐CODOX‐M/R‐IVAC was also effective in the BL‐like patients that were reclassified to DLBCL/HGBL in this study and therefore may be effective in borderline or atypical BL cases.

In summary, this prospective trial confirms that the rituximab with CODOX‐M/IVAC is deliverable, safe, and effective, with encouraging survival rates in a high‐risk BL cohort. Our findings provide a benchmark for future studies and confirm that R‐CODOX‐M/R‐IVAC should remain a recommended regimen for the treatment of BL. Randomised trials are needed to determine the optimum immunochemotherapy approach in BL. An international randomised trial comparing R‐CODOX‐M/R‐IVAC with lower intensity dose‐adjusted EPOCH‐R therapy is currently underway (EudraCT:2013‐004394‐27).

## AUTHOR CONTRIBUTIONS

AM, SR, RPe, RPa and DCL designed the study. AM, CB, SR, RPa, RPe, KMA, SM, SP and DCL recruited patients and provided data. CB and SB performed central pathology review. EHP, AK and AM analysed the data and wrote the manuscript. AL and LCH contributed to data collection and analysis. All authors critically reviewed the manuscript and approved the final version of the manuscript for submission.

## CONFLICT OF INTEREST

EHP has received research funding from F. Hoffman‐La Roche. KMA has received funding for travel expenses and medical advisory board participation from F. Hoffman‐La Roche. AKM has received honoraria from F. Hoffman‐La Roche and Amgen. All other authors declare that there is no conflict of interest.
